# Vortex-Induced Alignment of a Water Soluble Supramolecular Nanofiber Composed of an Amphiphilic Dendrimer

**DOI:** 10.3390/molecules18067071

**Published:** 2013-06-17

**Authors:** Taiki Yamamoto, Akihiko Tsuda

**Affiliations:** Department of Chemistry, Graduate School of Science, Kobe University, 1-1 Rokkodai-cho, Nada-ku, Kobe 657-8501, Japan

**Keywords:** dendrimer, naphthalene imide, amphiphilic molecule, supramolecular nanofiber, vortex, linear dichroism

## Abstract

We have synthesized a novel amphiphilic naphthalene imide bearing a cationic dendrimer wedge (NID). NID molecules in water self-assemble to form a two-dimensional ribbon, which further coils to give a linear supramolecular nanofiber. The sample solution showed linear dichroism (LD) upon stirring of the solution, where NID nanofibers dominantly align at the center of vortex by hydrodynamic interaction with the downward torsional flows.

## 1. Introduction

Small molecules cannot orient along the macroscopic flowing direction of the solvent molecules because at the molecular level Brownian motions are dominant over hydrodynamic interactions. However, when molecules or molecular assemblies are large enough to average local effects of the Brownian motions of the solvent molecules, they come to be able to sense fluidic flows. Actually, certain nanoscale molecules and molecular assemblies, having anisotropic structures, have been known to be affected by shear forces of fluids [1,2,3,4,5,6,7,8,9,10,11,12]. Ribó *et al*. have previously reported that electrostatic *J*-aggregates of (4-sulfophenyl)porphyrin derivatives, prepared in aqueous media with rotary stirring, become optically active, and suggested that hydrodynamic selection of either the right- or left-handed helical conformation of the aggregates is responsible [5,6]. In fact, the formation of helical ribbons in such a stirred solution has recently been confirmed. However, hydrodynamic behaviors of the aggregates in the dynamic fluids have not sufficiently been clarified experimentally. With this in mind, we recently succeeded in spectroscopic visualizations of macroscopic helical alignments of supramolecular polymers of *J*-aggregated zinc porphyrin, and anthracene derivatives in torsional flows of the vortex by mechanical rotary stirring of a fluid in an optical cell [7,8]. The results suggest that the supramolecular nanofibers can sense weak shear forces generated in the macroscopic fluid flows. Such vortex-induced alignment is observed not only in the supramolecular nanofibers, but also bionanofibers, such as DNA. In a vortex generated by mechanical rotary stirring in an optical cell, double-stranded DNA molecules in pure water also align helically to the spiral flow [12]. However, such vortex-induced alignment has not been achieved in water with an artificial supramolecular assembly. In this study we report that our newly designed supramolecular nanofiber, composed of an amphiphilic naphthalene imide derivative bearing a cationic dendrimer wedge (NID), showed vortex-induced alignment in water.

## 2. Results and Discussion

### 2.1. Synthesis of NID

**Scheme 1:**
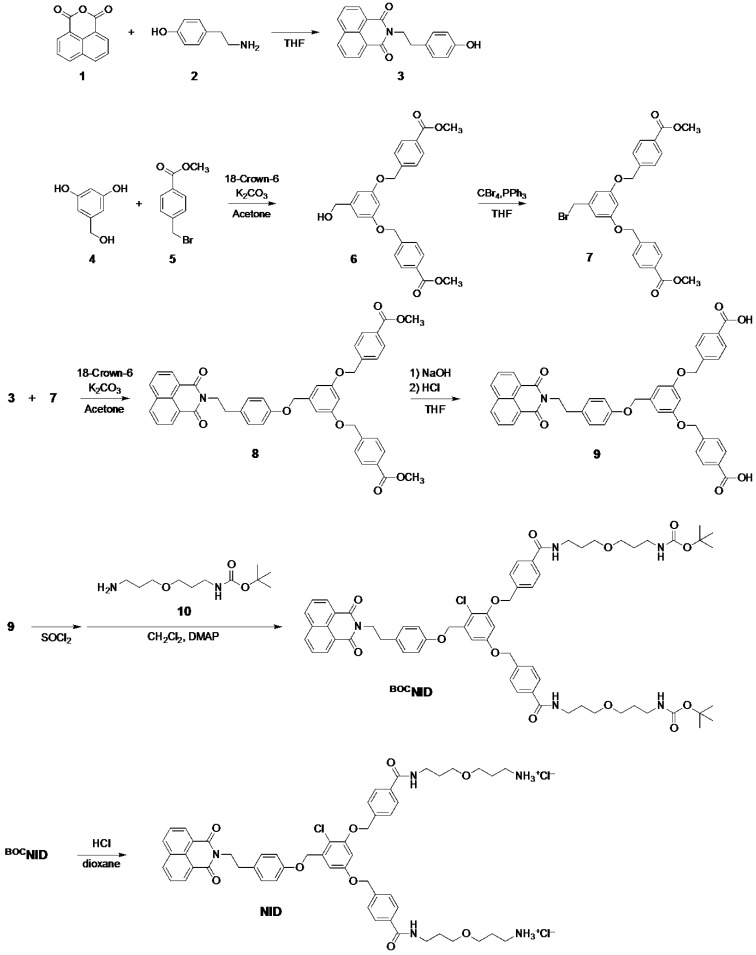
Synthetic procedures of NID.

NID was synthesized starting from 1,8-naphthalic anhydride (**1**), which was successfully converted to *N*-substituted naphthalene imide **3** in 43% yield through the reaction with 4-(2-aminoethyl)phenol (**2**) (Scheme 1) [13]. The coupling reaction of **3** with a dendron unit **7** gave a 75%yield of naphthalene imide dendrimer **8** [14,15], which was subsequently hydrolyzed to quantitatively form **9** having carboxyl groups. Then, substitution reactions of **9** with **10** provided *^BOC^*NID, which is highly soluble in organic solvents, with a 40% yield. Deprotections of BOC groups from *^BOC^*NID with HCl provided quantitatively an amphiphilic NID [16,17,18,19]. The molecular structure of NID was characterized by means of NMR, IR, and electronic absorption spectroscopy, together with mass spectrometry.

### 2.2. Self-Assembly of NID Molecules in Water

**Figure 1:**
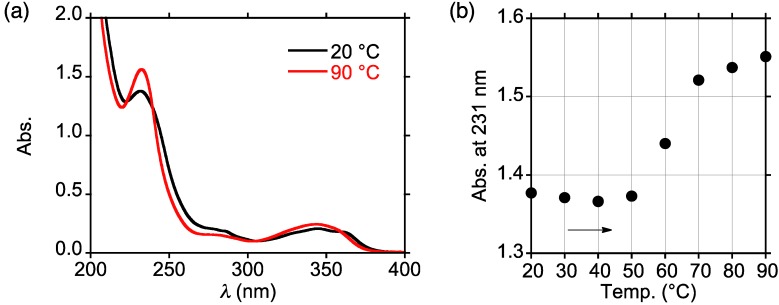
(**a**) Absorption spectra of NID in water at 20 °C and 90 °C. (**b**) Plot of the absorbance changes at 231 nm upon elevating temperature from 20 °C to 90 °C. [NID] = 2.5 × 10^−5^ M.

**Figure 2:**
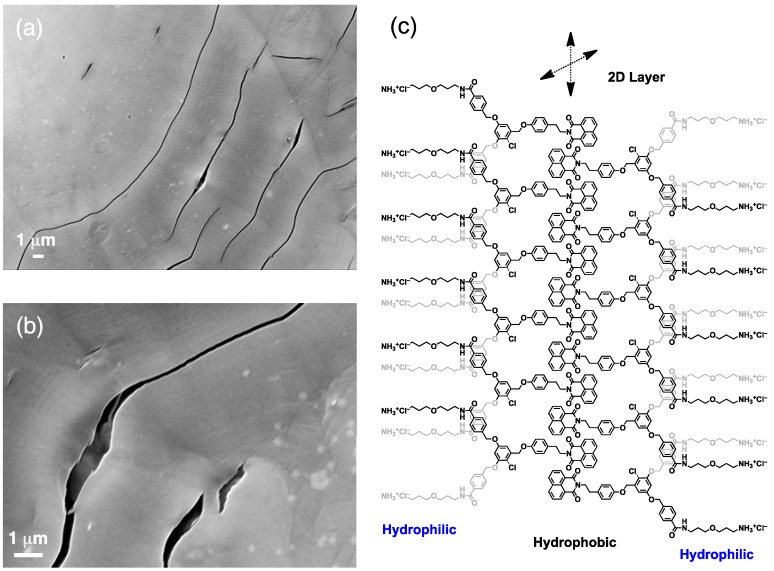
(**a**) and (**b**) Scanning electron micrographs of an air-dried sample of NID fibers prepared from a water solution of [NID] = 2.5 × 10^−5^ M deposited on a silicon substrate. (**c**) Schematic illustration of a possible self-assembling structure of NID.

In the absorption spectroscopy, a water solution of NID at 20 °C with a concentration of 2.5 × 10^−5^ M displayed broad absorption bands in the UV region (Figure 1a). However, upon heating of the sample solution to 90 °C, an absorption band of NID with *λ*_max_ = 231 nm became sharp, and further, the lower energy band was blue shifted into the 300–370 nm range. A plot of the absorbance changes at 231 nm upon elevating the temperature shows that the notable spectral change occurs from 50 °C and plateau at 90 °C (Figure 1b). The observed spectral change most likely originates from thermal dissociation of the aggregate. Scanning electron microscopy (SEM), performed on a silicon substrate with a solution of self-assembled NID in water, after being air-dried under flowing air, revealed the formation of linear fibers, which aligned along the flowing direction of the air (Figure 2). The observed fibers seem to coil in a thin-layer, like a paper string (Figure S1). The amphiphilic NID molecules in a water solvent may self-assemble via the hydrophobic interactions and ionic interactions to form the sheet structure [20], where naphthalene imide components may assemble through π-stacking interaction with a slipped parallel arrangement judging from the red-shifted absorption bands of the assembled structure at lower temperature (Figure 1a and Figure 2c). The formed thin sheet may then roll up to form the fibrous structure. The shear-induced alignments at air–water and/or water–substrate interfaces of flowing air and water resulted in the observed alignment of the fibers.

### 2.3. Vortex-Induced Alignment of NID Fibers

**Figure 3:**
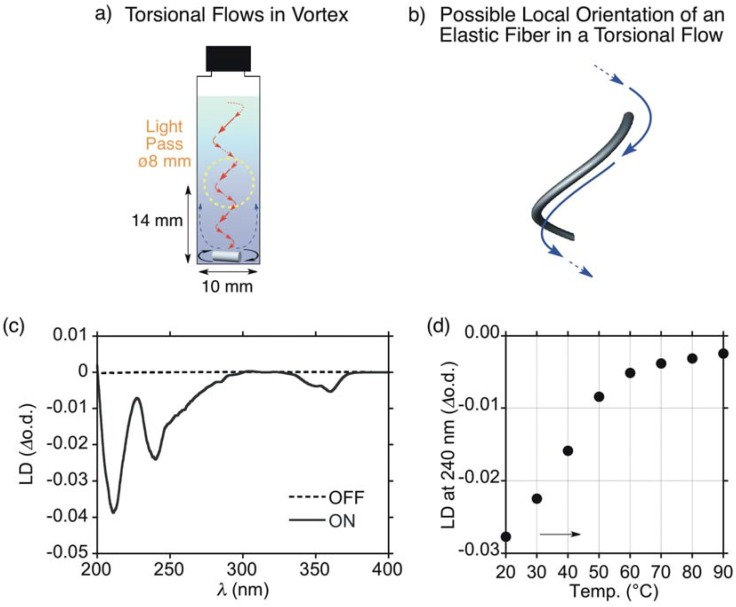
(**a**) Schematic illustration of a sample solution with rotary stirring in a 10 × 10 × 40 mm quartz optical cuvette containing a magnetic stir bar. For LD spectroscopy, an *φ*8.0 mm wide linear polarized light is allowed to pass through the sample solution at a position 14 mm above the bottom of the inner surface of the optical cuvette. (**b**) Vertical helical alignment of a fiber in a torsional flow. (**c**) LD spectroscopy for a water solution of NID nanofibers with or without rotary stirring at 20 °C. (**d**) Plot of the LD intensity changes at 240 nm upon elevating temperature from 20 °C to 90 °C. [NID] = 2.5 × 10^−5^ M. Stirring: 1350 rpm using a *φ*2.0 × 5.0 mm Teflon-coated magnetic stirring bar.

With these structural features of NID assemblies in mind, we have investigated the hydrodynamic behavior of the formed supramolecular nanofiber in a torsional vortex flow. Orientation of the NID nanofiber in a water solution can be characterized by using LD spectroscopy [21,22]. The spectrometer was equipped with a 10 × 10 × 40 mm quartz optical cuvette, in which sample solutions (3 mL) were stirred mechanically using a *φ* 2.0 × 5.0 mm Teflon-coated magnetic stir bar at the bottom of the cuvette, 13 mm below the center of a *φ*8.0 mm wide polarized light beam (Figure 3a) [8]. While a water solution of NID (2.5 × 10^−5^ M) without stirring was LD silent, it became LD active upon mechanical rotary stirring (Figure 3c). 

When the solution containing nanofibres, composed of NID, was stirred at 1,350 rpm, it displayed intense LD bands at the absorption bands of the naphthalene component [360 (Δo.d. = −0.005), 240 (−0.024), and 211 nm (−0.039)]. This accord with other cases, where supramolecular nanofibers showed vortex-induced LD in organic solvents [7,8,9]. NID fibers in water most likely align vertically along the downward torsional flow at the center of vortex (Figure 3b). Expectedly, the observed LD intensity decreased as the temperature elevated and became silent at 90 °C due to dissociation of the assemblies (Figure 3c). However, in contrast to the profile of temperature dependent absorption spectral change (Figure 1b), which showed a slight decrease, and then a large increase of the absorbance upon elevating temperature from 20 to 50 °C and from 50 to 90 °C, respectively, this LD profile showed a large change of the intensity in the lower temperature region. By reference to the vortex-induced alignment of DNA in water, where double-stranded DNA allowed the vortex-induced alignment, but its dissociated single-stranded form hardly showed such behavior [12], structural growth of the NID assemblies into the nanofiber form, occurring dominantly at the lower temperature region, may allow its subsequent hydrodynamic alignment in the vortex flows.

## 3. Experimental

### 3.1. Measurements

UV-Vis spectra were recorded on a JASCO type V-570 UV/VIS/NIR spectrometer equipped with a JASCO type PTC-505T temperature/stirring controller. LD spectra were recorded on a JASCO J-820 spectropolarimeter equipped with a JASCO PTC-423L temperature/stirring controller. ^1^H (500 MHz) and ^13^C-NMR (125 MHz) spectra were recorded on a Bruker AVANCE 500 spectrometer. Chemical shifts (*δ* in ppm) are reported with respect to tetramethylsilane as the internal standard. Infrared absorption spectra (IR) were recorded on a JASCO FT/IR-4200 Fourier transform infrared spectrometer. FAB mass spectrometry was performed on a JEOL JMS-BU30 LC Mate spectrometer with 3-nitrobenzylalcohol as the matrix.

### 3.2. Synthesis

*2-[2-(4-Hydroxyphenyl)ethyl]-1H-benz[de]isoquinoline-1,3(2H)-dione* (**3**). A dry THF solution (10 mL) containing a mixture of 1,8-naphthalic anhydride (**1**, 0.50 g, 2.5 mmol) and tyramine (**2**, 0.34 g, 2.5 mmol) was refluxed for 1 day under Ar. The reaction mixture was evaporated to dryness and the residue was washed with acetone. The residue was filtrated and evaporated to dryness to give **3** as pale brown solid in 43% yield (0.34 g, 1.08 mmol). FAB-MS *m/z* calcd for M^+^ (C_20_H_15_NO_3_) 317, found 318 (M^+^+1); ^1^H-NMR (acetone-*d_6_*, 20 °C): *δ* (ppm) 8.57 (d, 2H, *J* = 7.3 Hz, ArH), 8.44 (d, 2H, *J* = 7.2 Hz, ArH), 7.89 (t, 2H, *J* = 7.2 Hz, ArH), 7.17 (d, 2H, *J* = 8.5 Hz, ArH), 6.79 (d, 2H, *J* = 8.5 Hz, ArH), 4.32–4.29 (m, 2H, benzyl), 2.94–2.91 (m, 2H, alkyl); ^13^C-NMR (CDCl_3_, 20 °C): *δ* (ppm) 165.13, 157.56, 135.70, 133.48, 132.31, 131.38, 129.61 128.73, 124.48, 116.85, 43.26, 34.73; IR (KBr): 3391, 1695, 1647, 1620, 1590, 1594, 1515, 1449, 1392, 1355, 1261, 1234, 1174, 775 cm^−^^1^.

*4,4'-[[5-[[4-[2-(1,3-Dioxo-1H-benzo[de]isoquinolin-2(3H)-yl)ethyl]phenoxy]methyl]-1,3-phenylene]bis(oxymethylene)]bis-benzoic acid, dimethyl ester* **8**. An acetone solution (6 mL) containing a mixture of **3** (0.16 g, 0.50 mmol), **7** (0.25 g, 0.50 mmol), anhydrous K_2_CO_3_ (0.09 g, 0.63 mmol), and 18-crown-6 (0.02 g, 0.05 mmol), was refluxed for 1 day under Ar. The reaction mixture was evaporated to dryness. The solid residue was dissolved in CH_2_Cl_2_, and the solution was washed with water and brine. The organic layer was extracted and dried over anhydrous Na_2_SO_4_, and then evaporated to dryness. The residue was recrystallized with CH_2_Cl_2_/methanol to give **8** as white solid in 75% yield (0.24 g, 0.38 mmol). FAB-MS *m/z* calcd for M^+^ (C_45_H_37_NO_9_) 736, found 736 (M^+^); ^1^H-NMR (CDCl_3_, 20 °C): *δ* (ppm) 8.61 (d, 2H, *J* = 7.2 Hz, ArH), 8.22 (d, 2H, *J* = 7.2 Hz, ArH), 8.05 (d, 4H, *J* = 8.5 Hz, ArH), 7.77 (t, 2H, *J* = 7.2 Hz, ArH), 7.49 (d, 4H, *J* = 8.5 Hz, ArH), 7.29 (d, 2H, *J* = 8.5 Hz, ArH), 6.90 (d, 2H, *J* = 8.5 Hz, ArH), 6.69 (d, 2H, *J* = 2.4 Hz, ArH), 6.54 (t, 1H, *J* = 2.4 Hz, ArH), 5.10 (s, 4H, benzyl), 4.98 (s, 2H, benzyl), 4.38–4.35 (m, 2H, benzyl), 3.93 (s, 6H, methoxy), 2.99–2.96 (m, 2H, alkyl); ^13^C-NMR (CDCl_3_, 20 °C): *δ* (ppm) 166.98, 164.24, 159.99, 157.41, 142.09, 140.05, 138.70, 134.13, 131.76, 131.37, 130.18, 130.04, 129.86, 128.33, 127.16, 127.12, 122.82, 115.01, 106.57, 101.72, 69.94, 69.56, 52.31, 42.13, 33.56; IR (KBr): 3423, 2950, 1510, 1436, 1415, 1384, 1343, 1235, 1108, 1067, 1019, 844, 781, 756 cm^−1^.

*4,4'-[[5-[[4-[2-(1,3-Dioxo-1H-benzo[de]isoquinolin-2(3H)-yl)ethyl]phenoxy]methyl]-1,3-phenylene]bis(oxymethylene)]bis-benzoic acid* **9**. To a THF (20 mL) solution of **8** (100 mg, 0.14 mmol) was added an aqueous solution of NaOH (0.6 M, 20 mL), and the mixture was stirred for 12 h at 50 °C. Then, the reaction mixture was neutralized with HClaq. The solvent was evaporated to dryness. The residue was washed with water, and dried in vacuo to give **9** as white solid in 78% yield (74 mg, 0.11 mmol). ^1^H-NMR (DMSO-*d*_6_, 20 °C): *δ* (ppm) 13.03 (s, 2H, COOH), 8.54 (d, 2H, *J* = 7.7 Hz, ArH), 8.50 (d, 2H, *J* = 7.7 Hz, ArH), 7.99 (d, 4H, *J* = 8.5 Hz, ArH), 7.91 (t, 2H, *J* = 7.7 Hz, ArH), 7.59 (d, 4H, *J* = 8.5 Hz, ArH), 7.22 (d, 4H, *J* = 8.5 Hz, ArH), 6.95 (d, 2H, *J* = 8.5 Hz, ArH), 6.75 (d, 2H, *J* = 2.4 Hz, ArH), 6.69 (t, 1H, *J* = 2.4 Hz, ArH), 5.22 (s, 4H, benzyl), 5.03 (s, 4H, benzyl), 4.25 (m, 2H, benzyl), 2.90 (m, 2H, alkyl); ^13^C-NMR (DMSO-*d*_6_, 20 °C): *δ* (ppm) 168.06, 164.27, 160.32, 157.73, 142.97, 140.75, 135.34, 132.29, 131.73, 131.10, 130.60, 130.44, 128.37, 128.23, 123.02, 115.79, 107.52, 69.84, 69.66, 42.17, 33.62.

***^BOC^*NID**. Compound **9** (87 mg, 0.12 mmol) was dissolved in thionyl chloride (10 mL), and the solution was refluxed under Ar for 4 h. The reaction mixture was evaporated to dryness. The residue was mixed with *tert*-butyl (3-(3-aminopropoxy)propyl)carbamate (**10**, 114 mg, 0.49 mmol) and *N*,*N*′-dimethyl-4-aminopyridine (60 g, 0.49 mmol) in dry CH_2_Cl_2_ (10 mL), and the solution was refluxed under Ar for 24 h. The reaction mixture was evaporated to dryness. The residue was dissolved into CHCl_3_, and washed with aqueous solutions of 1 N HCl and saturated NaHCO_3_. The organic layer was extracted, dried over anhydrous Na_2_SO_4_, and evaporated to dryness. The residue was chromatographed on silica gel with ethylacetate as eluent, where the second fraction was collected and evaporated to leave *^BOC^*NID as white solid in 40% yield (56 mg, 0.05 mmol). FAB-MS *m/z* calcd for M^+^ (C_65_H_76_ClN_5_O_13_) 1169, found 1170 (M^+^+1); ^1^H-NMR (CDCl_3_, 20 °C): *δ* (ppm) 8.61 (d, 2H, *J* = 7.2 Hz, ArH), 8.23 (d, 2H, *J* = 8.5 Hz, ArH), 7.82–7.75 (m, 6H, ArH), 7.46 (d, 2H, *J* = 8.5 Hz, ArH), 7.37 (d, 2H, *J* = 8.5 Hz, ArH), 7.30 (d, 2H, *J* = 8.5 Hz, ArH), 7.17 (br, 2H, NH), 6.93 (d, 2H, *J* = 8.5 Hz, ArH), 6.84 (d, 1H, *J* = 2.4 Hz, ArH), 6.54 (d, 1H, *J* = 2.4 Hz, ArH), 5.16 (s, 2H, benzyl), 5.13 (s, 2H, benzyl), 5.04 (s, 2H, benzyl), 4.91 (br, 2H, NH), 4.38 (m, 2H, benzyl), 3.60 (br, 4H, alkyl), 3.56 (t, 4H, *J* = 5.3 Hz, alkyl), 3.49 (t, 4H, *J* = 5.3 Hz, alkyl), 3.23 (br, 4H, alkyl), 2.98 (m, 2H, alkyl), 1.91 (br, 4H, alkyl), 1.74 (m, 4H, alkyl), 1.41 (s, 18H, BOC); ^13^C-NMR (CDCl_3_, 20 °C): *δ* (ppm) 167.20, 167.07, 164.12, 157.93, 157.81, 157.08, 156.12, 154.56, 139.80, 137.81, 137.23, 134.54, 134.02, 131.63, 131.60, 130.10, 128.19, 127.40, 127.34, 127.24, 126.99, 126.85, 122.85, 114.94, 113.31, 106.32, 101.45, 70.31, 69.76, 69.61, 69.48, 68.65, 68.57, 67.22, 41.97, 38.39, 38.30, 37.92, 33.44, 30.15, 29.22, 28.42

**NID**. To a 1,4-dioxane (4.0 mL) solution of *^BOC^*NID (20 mg, 17 μmol) was added an aqueous solution of HCl (8.0 M, 4.0 mL), and the reaction mixture was stirred for 3 h at 50 °C. The reaction mixture was evaporated to dryness, and the residue was washed with acetone. The residue was dried in vacuo to give NID as white solid in 80% yield (14 mg, 13 μmol). FAB-MS *m/z* calcd for M^+^ (C_55_H_60_ClN_5_O_9_) 969, found 970 (M^+^+1); ^1^H-NMR (DMSO-*d*_6_, 20 °C): *δ* (ppm) 8.60 (t, 2H, *J* = 5.4 Hz, NH), 8.55 (d, 2H, *J* = 7.2 Hz, ArH), 8.52 (d, 2H, *J* = 8.5 Hz, ArH), 7.94–7.89 (m, 6H, ArH), 7.59 (d, 2H, *J* = 8.5 Hz, ArH), 7.55 (d, 2H, *J* = 8.5 Hz, ArH), 7.25 (d, 2H, *J* = 8.5 Hz, ArH), 6.99–6.98 (m, 3H, ArH), 6.91 (d, 1H, *J* = 2.4 Hz, ArH), 5.33 (s, 2H, benzyl), 5.21 (s, 2H, benzyl), 5.11 (s, 2H, benzyl), 4.26 (m, 2H, benzyl), 3.49–3.43 (br, 12H, alkyl), 2.91 (br, 6H, alkyl), 1.80 (br, 8H, alkyl); ^13^C-NMR (DMSO-*d*_6_, 20 °C): *δ* (ppm) 166.88, 166.81, 164.22, 158.51, 157.60, 155.28, 149.59, 146.60, 140.55, 140.43, 137.29, 135.31, 135.13, 135.02, 132.24, 132.17, 131.67, 130.63, 128.31, 128.26, 128.17, 128.03, 123.14, 115.69, 113.62, 108.29, 70.08, 68.64, 67.93, 67.79, 42.22, 37.57, 37.26, 33.57, 30.28, 28.11; IR (KBr): 3398, 2930, 1698, 1544, 1384, 1342, 1235, 1170, 1100, 1032, 845, 780, 755 cm^−^^1^.

## 4. Conclusions

We have successfully synthesized a newly designed amphiphilic naphthalene imide derivative bearing a cationic dendrimer wedge (NID). NID is soluble in water, and self-assembles to form a two-dimensional ribbon, which further coils to be a fibrous supramolecular assembly of nanoscale diameter. The sample solution showed induced linear dichroism (LD) upon stirring of the solution, where NID nanofibers vertically aligned at the center of the vortex flow. The present study showed spectroscopically the hydrodynamic behavior of an artificial water-soluble nanofiber in a vortex flow. The results suggest a new insight into the future controls of molecular dynamics and chemical reactions in water.

## Supplementary Materials

Supplementary materials can be accessed at: http://www.mdpi.com/1420-3049/18/6/7071/s1.
