# Supplementary material for: Vortex-Induced Alignment of a Water Soluble Supramolecular Nanofiber Composed of an Amphiphilic Dendrimer
Source: Molecules. 2013 Jun 17;18(6):7071–80. doi: 10.3390/molecules18067071 (PMC6270294; doi:10.3390/molecules18067071)

Supplementary Materials

1. A Model for Coiling of the Thin Layer Film

Figure S1. Formation of a paper string from a paper ribbon.

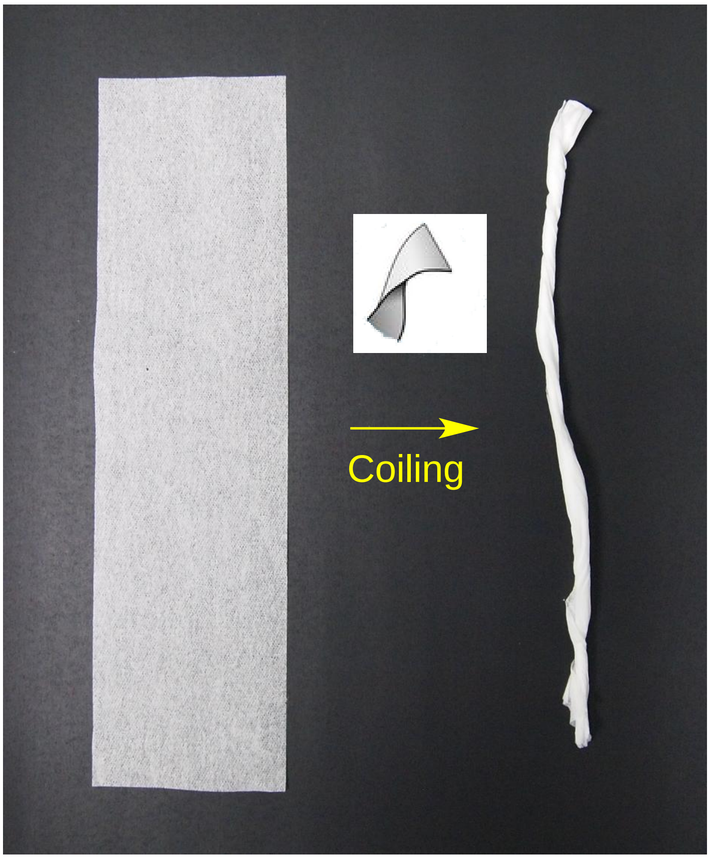

^{1}H and ^{13}C NMR Spectra of 3 in Acetone-d_6

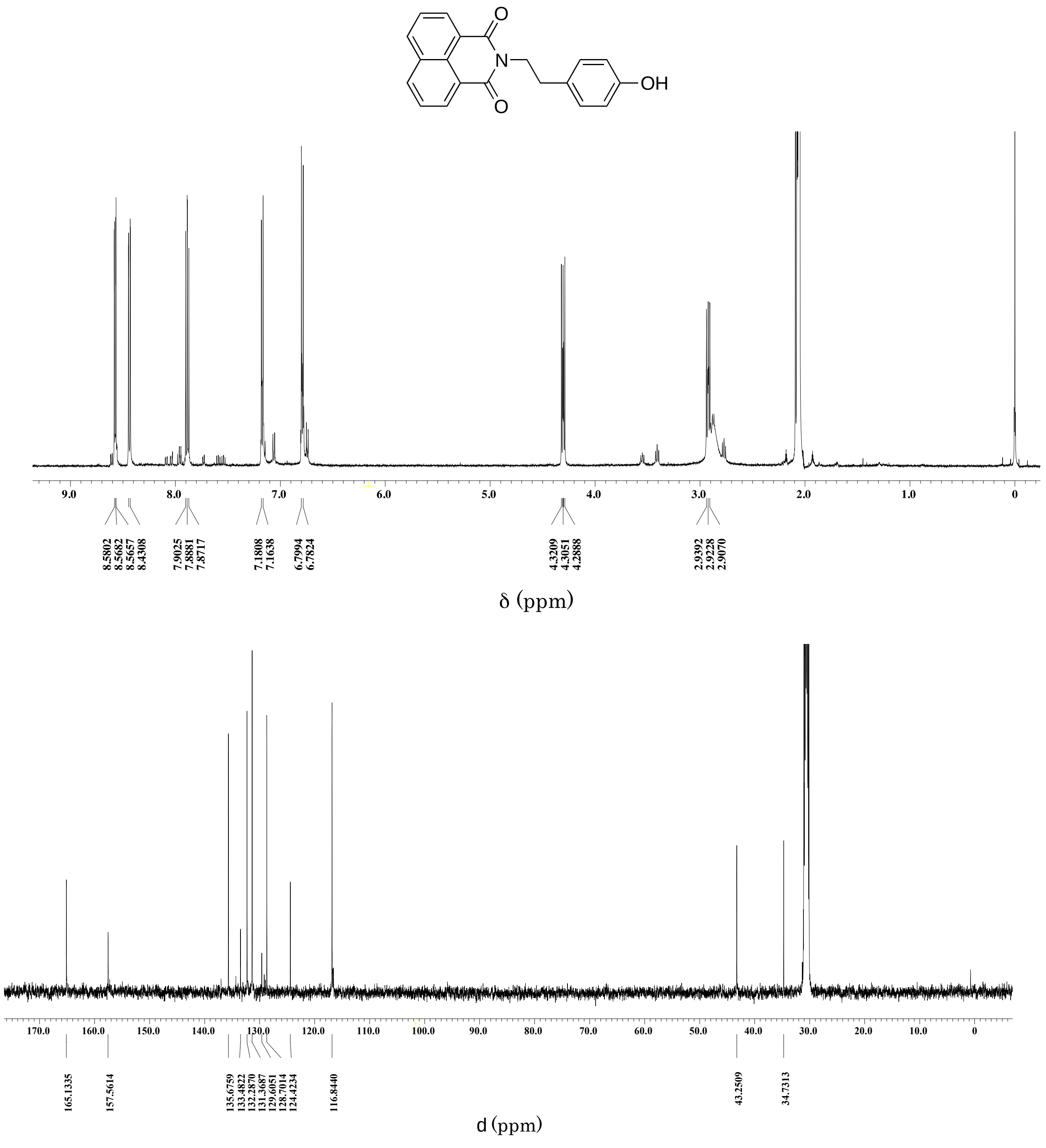

^{1}H and ^{13}C NMR Spectra of 8 in $CDCl_3$

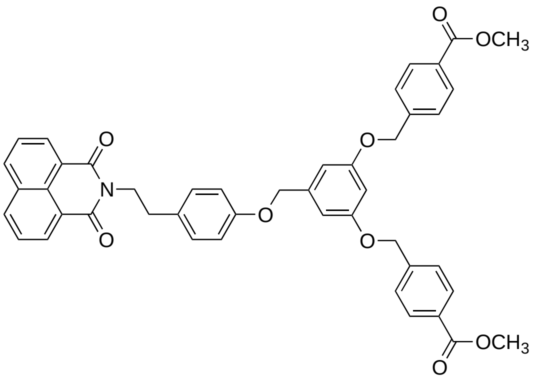

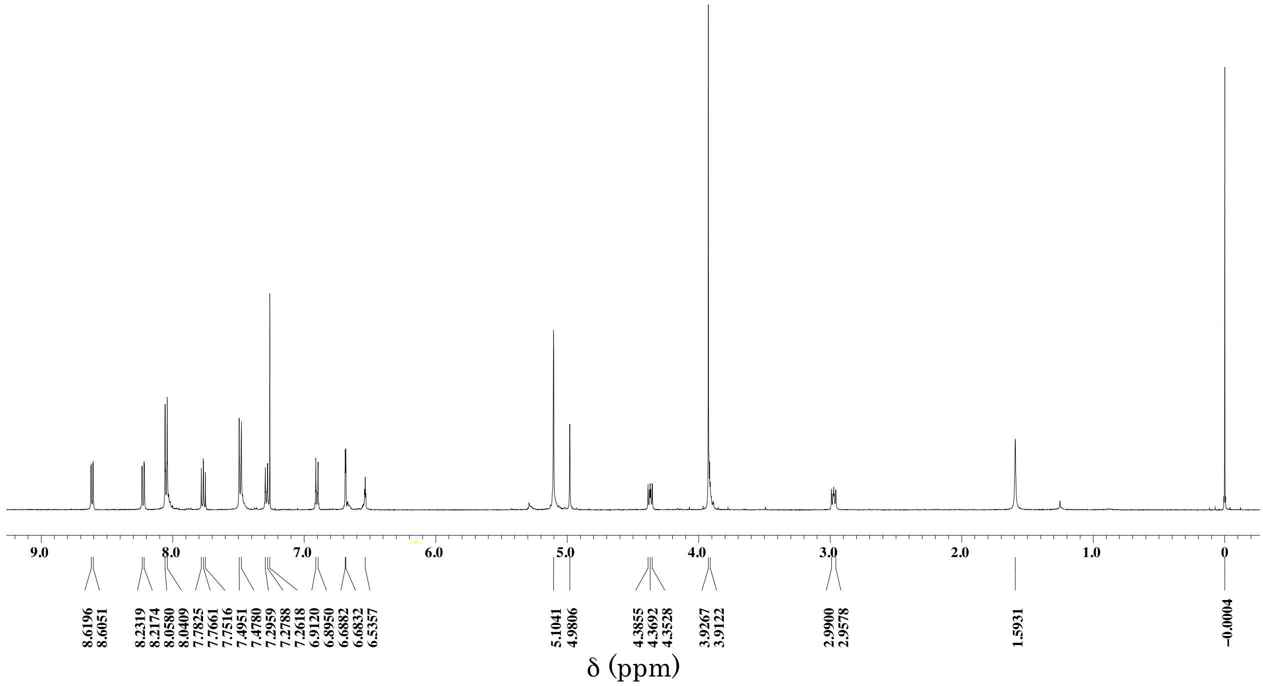

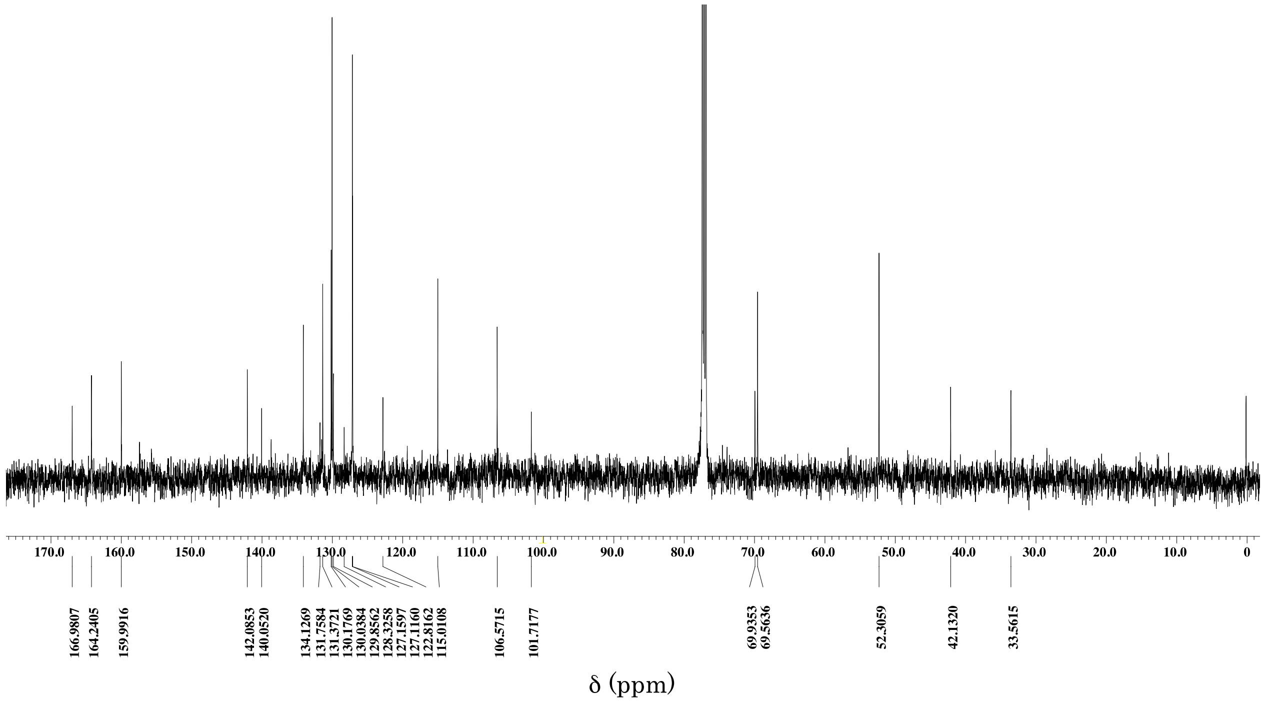

^{1}H and ^{13}C NMR Spectra of 9 in DMSO-d_6

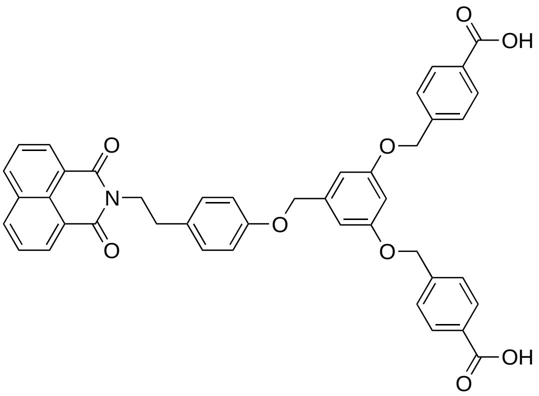

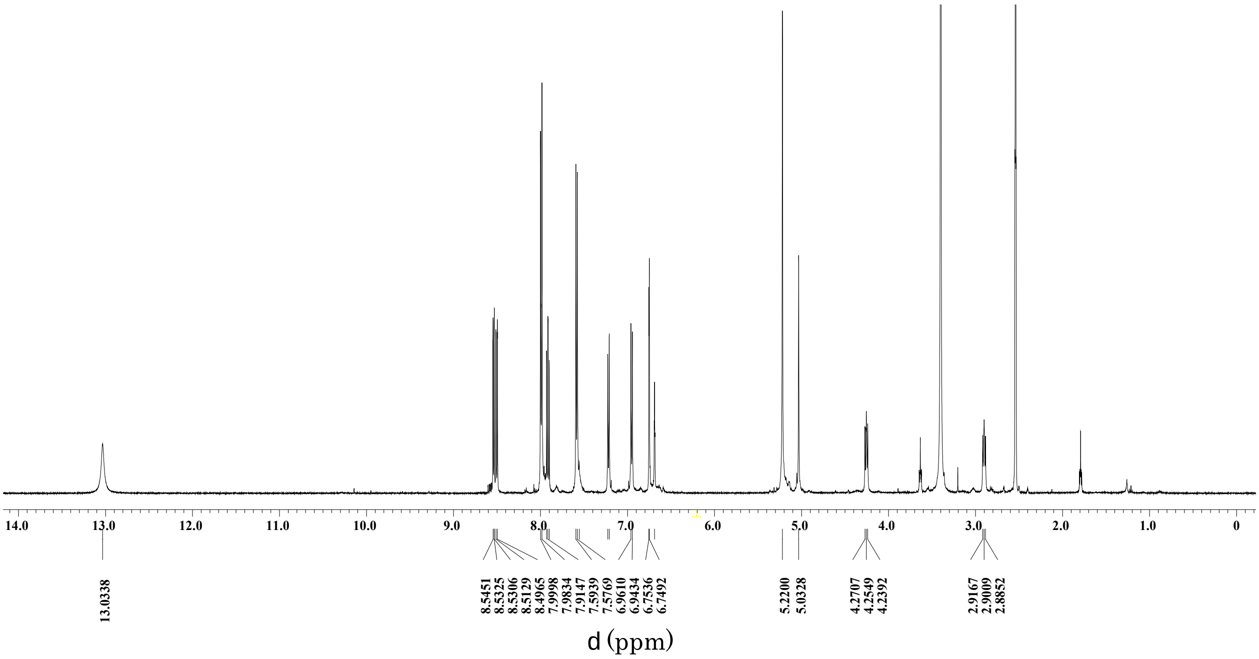

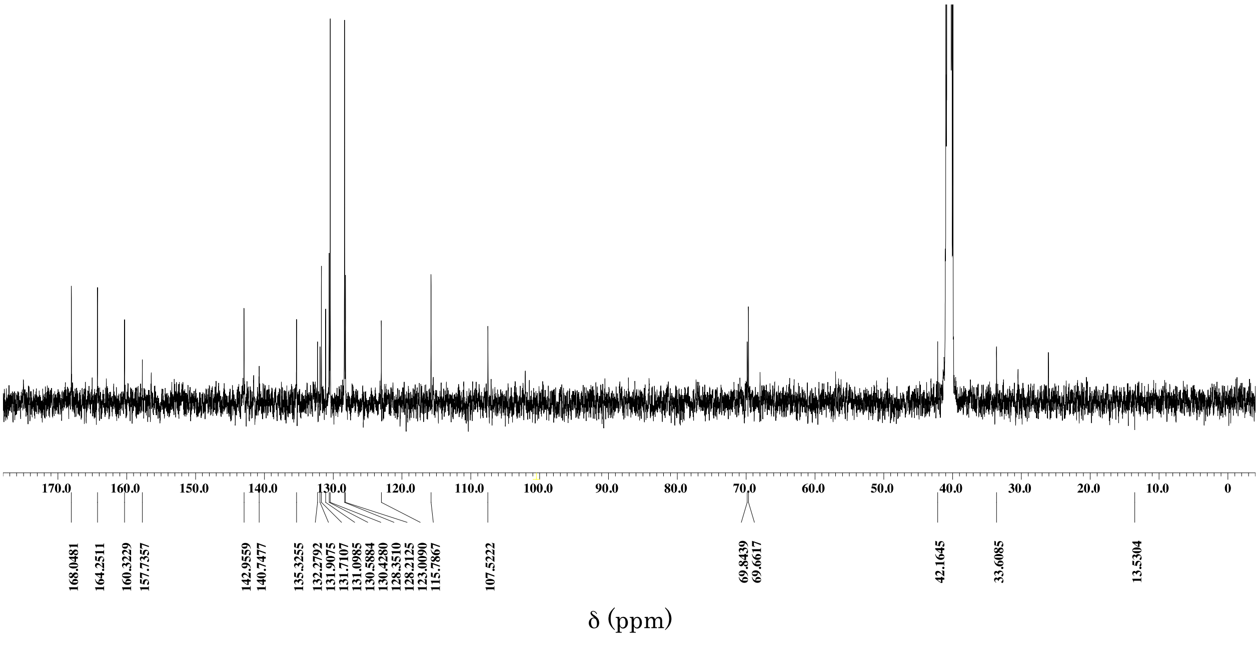

^{1}H and ^{13}C NMR Spectra of ^{BOC}NID in $CDCl_3$

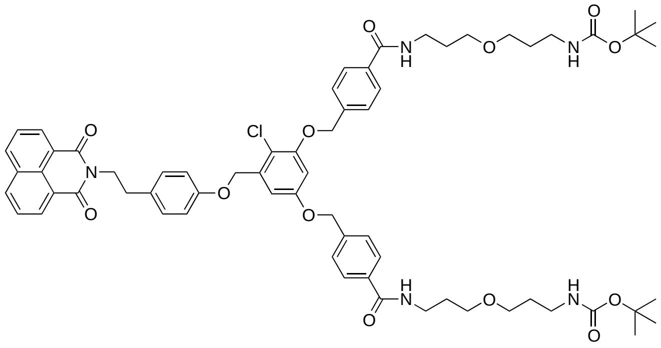

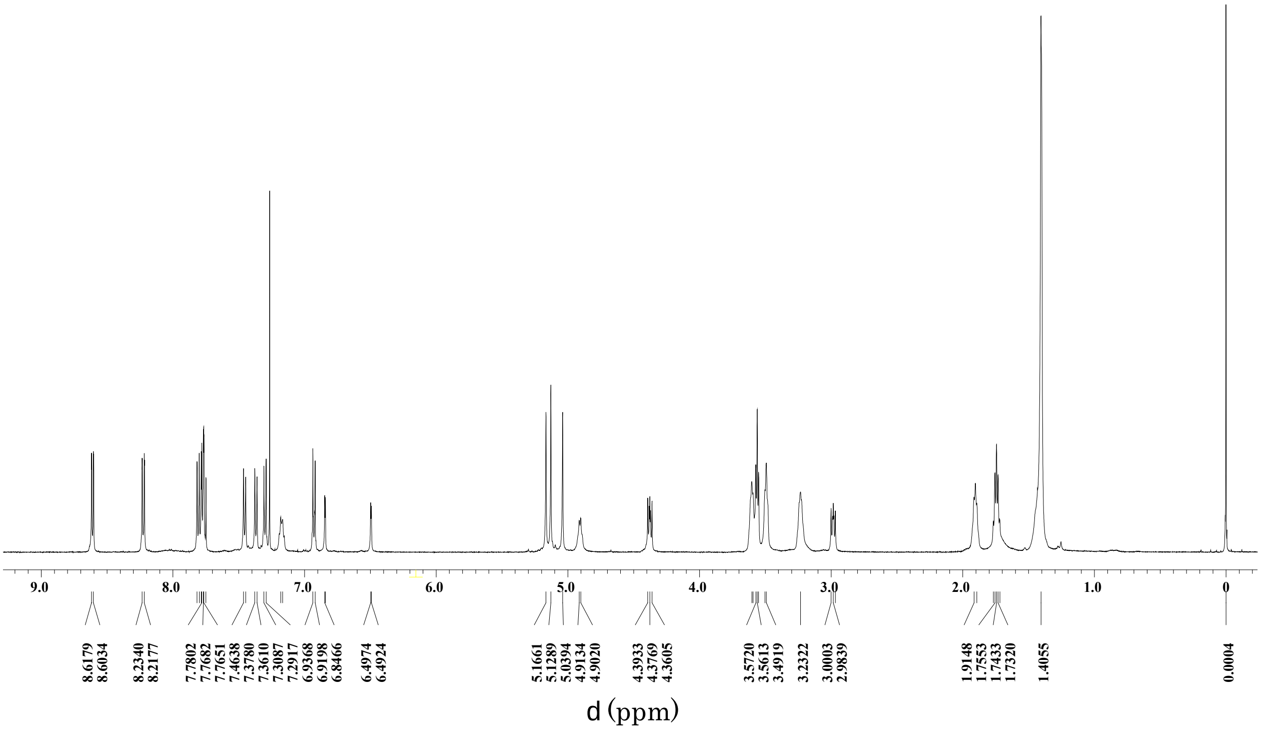

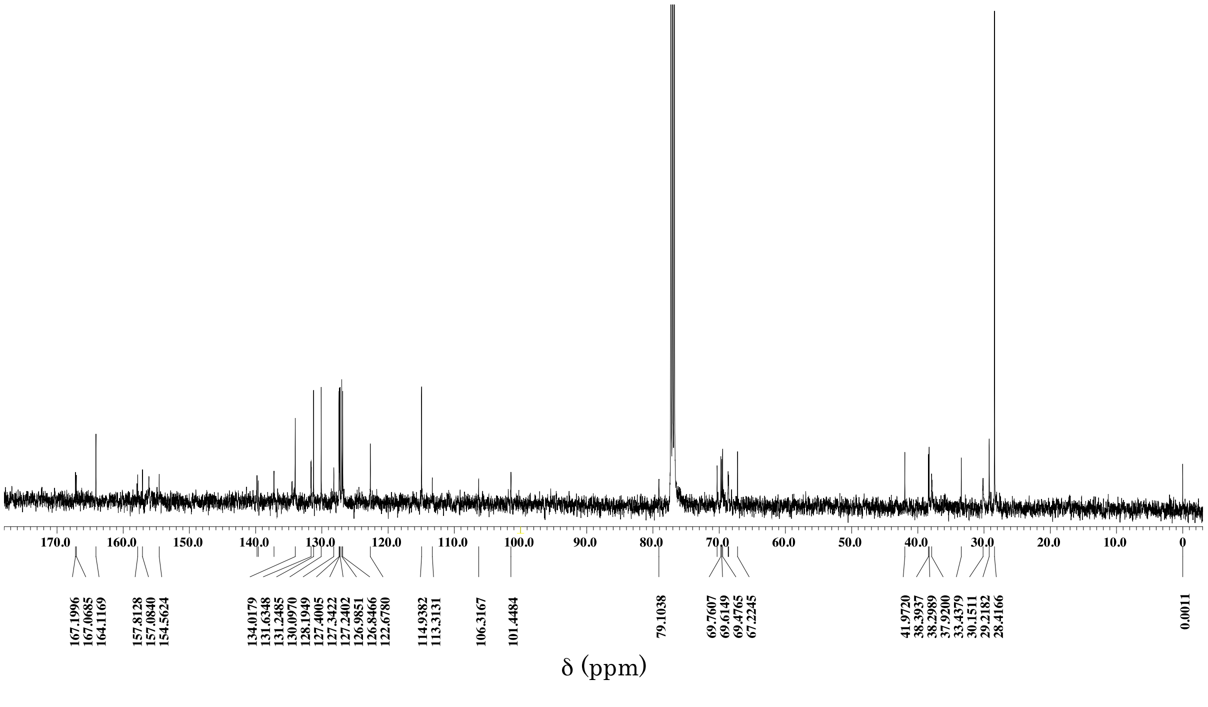

^{1}H and ^{13}C NMR Spectra of NID in DMSO-d_6

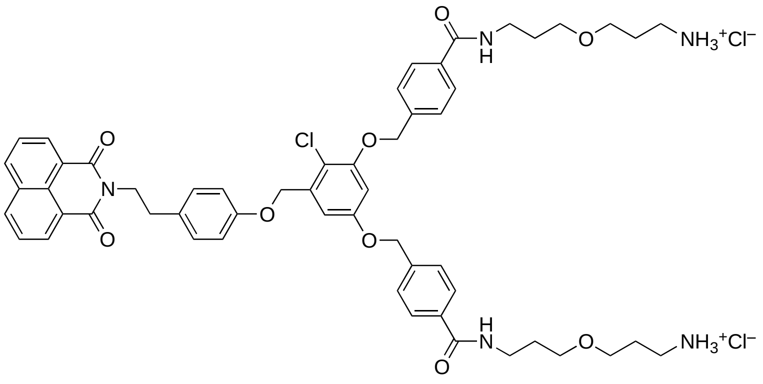

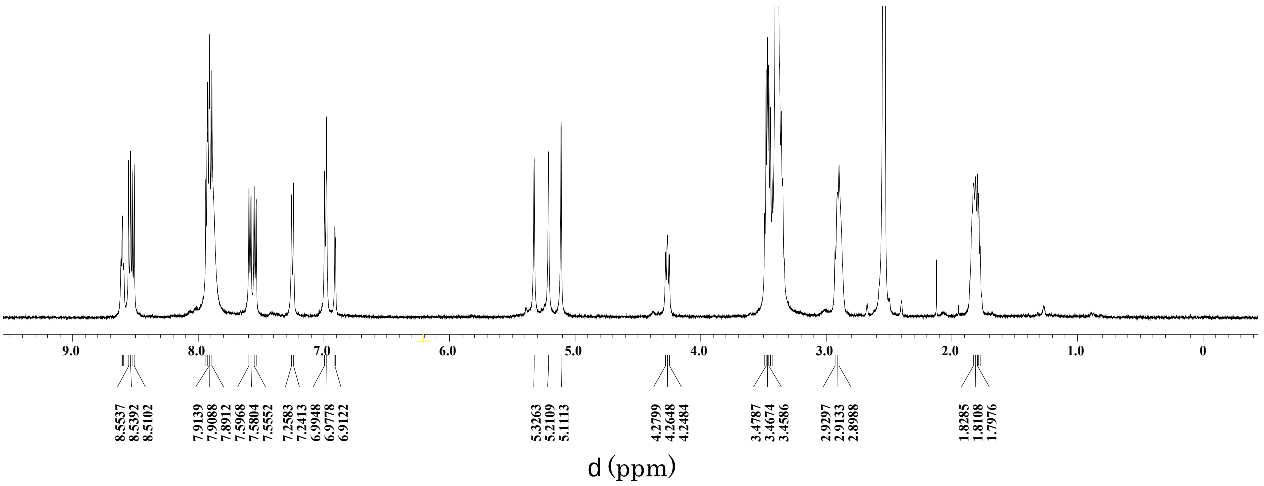

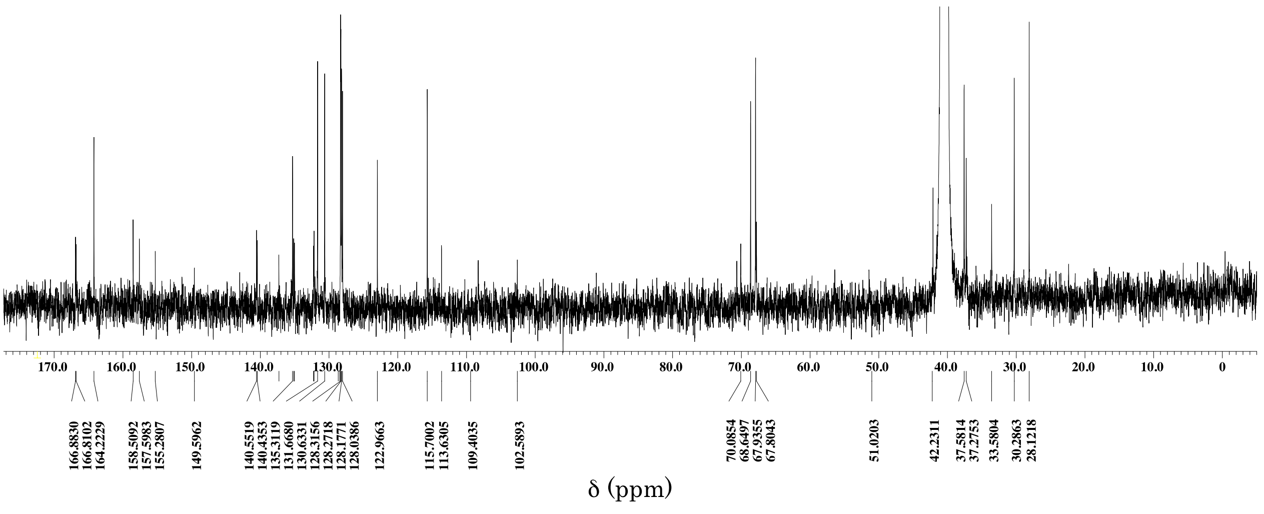

Supplement: Supplementary file 1 [file molecules-18-07071-s001.pdf]
